# The effects of gallic acid and metformin on male reproductive dysfunction in diabetic mice induced by methylglyoxal: An experimental study

**DOI:** 10.18502/ijrm.v19i8.9619

**Published:** 2021-09-09

**Authors:** Zeinab Behdarvand-Margha, Akram Ahangarpour, Mohammadreza Shahraki, Gholamreza Komeili, Layasadat Khorsandi

**Affiliations:** ^1^Department of Physiology, Faculty of Medicine, Zahedan University of Medical Sciences, Zahedan, Iran.; ^2^Department of Physiology, Faculty of Medicine, Physiology Research Center, Medical Basic Sciences Research Institute, Ahvaz Jundishapur University of Medical Sciences, Ahvaz, Iran.; ^3^Department of Anatomical Sciences, Faculty of Medicine, Cellular and Molecular Research Center, Medical Basic Sciences Research Institute, Ahvaz Jundishapur University of Medical Sciences, Ahvaz, Iran.

**Keywords:** Diabetes mellitus, Gallic acid, Male reproductive system, Metformin, Mice.

## Abstract

**Background:**

Diabetes mellitus is a disease that has reached a dangerous point. Today, nearly 500 million men and women around the world live with diabetes. Gallic acid (Gal) affects diabetes.

**Objective:**

To evaluate the effects of Gal and metformin (met) on the levels of glucose, insulin, testosterone, luteinizing hormone (LH), follicle-stimulating hormone (FSH), sperm count, antioxidant status, and histological changes in the testes of diabetic mice induced by methylglyoxal (MGO).

**Materials and Methods:**

In this experimental study, 50 male adult NMRI mice, weighting 25-30 gr, aged 3-4 months were randomly divided into five equal groups (n = 10/each). (i) Control (vehicle, normal saline), (ii) MGO (600 mg/kg/d) orally for 28 days, (iii) Gal (50 mg/kg/d), (iv) MGO+Gal, and (v) MGO+met (200 mg/kg/d). Gal and met were administered orally for 21 consecutive days after the induction of diabetes. Blood samples were taken at 24 hr after the latest doses of treatment. Histological assessment of the testis was done, and the epididymis sperm count was obtained. Antioxidant indices, glucose, insulin, LH, FSH, and testosterone levels were measured.

**Results:**

In the MGO group compared to the control group, insulin, glucose (p = 0.001), LH (p = 0.04) and malondialdehyde (p = 0.001) were increased. However, the level of testosterone (p = 0.001), seminiferous tubule diameters, epithelial height, sperm count, superoxide dismutase activity (p = 0.02), and testis volume (p = 0.01) were decreased. The results indicated that Gal and met ameliorated the MGO effects.

**Conclusion:**

These findings suggested that the animals receiving MGO became diabetic. According to the results, Gal and met can effectively prevent MGO-induced diabetes. The effect of Gal was equivalent and sometimes better than metformin.

## 1. Introduction

Diabetes mellitus (DM) is a disorder that is widely studied in terms of impaired glucose regulation, thus providing insights into glucose metabolism. Diabetes is a group of chronic and metabolic diseases that are caused by defects in insulin secretion or insulin function. DM has a negative effect on several organs of the body, and can harm the cardiovascular system, nerves, and kidneys, eyes, and male reproductive system, leading to degenerative changes in the testicles and epididymis, decrease in semen volume, sperm count and sperm motility, and changes in sperm morphology (1).

People with metabolic syndrome and DM may have reproductive problems, and they may experience depressed mood, lack of sexual desire, and erectile dysfunction. DM is known as a heterogeneous set of metabolic disorders characterized by the common hyperglycemic phenotype caused by impaired insulin secretion, function, or both (2). Glucose metabolism plays an important role in spermatogenesis and a previous study emphasized the hazardous effects of hyperglycemia on the male sexual function such as sperm parameters and therefore can affect oxidative stress indices, decreased Leydig cells, and secretion of testosterone and FSH (3). Methylglyoxal (MGO) is an organic compound and a reduced derivative of pyruvic acid. It is a reactive compound that is involved in the development of diabetes. Experimental studies have shown that the amount of MGO increase with hyperglycemia and aging. MGO may play a role in diabetes-induced vessel dysfunction and oxidative stress-induced apoptosis in endothelial cells (4). Dhar and colleagues reported that intravenous injection of MGO caused the induction of type-2 diabetes and pancreatic β-cells dysfunction in their study (5). Also, MGO is used for the induction of type-2II diabetes in rodents. Oxidative stress is involved in the pathophysiology and reproductive disorders of men associated with diabetes (6).

Gallic acid (Gal) a type of polyphenol and is commonly present in gallnuts, sumac, tea leaves, oak bark, different berries, and grapes. The chemical formula of Gal is C6H2(OH)3COOH. Pharmacological studies have suggested that Gal has anti-inflammatory, anti-coagulant, and anti-tumor effects (7). Gal, as a potent antioxidant, has antidiabetic properties (8). Oyagbemi and colleagues showed that GA can have a beneficial effect on testis cyclophosphamide toxicity in rats and can improve reproductive parameters (9).

Since the effects of Gal on reproductive function in MGO-induced diabetic rats have not been previously reported, the aim of this study was to investigate the effect of Gal on the testicular tissue, testosterone, luteinizing hormone (LH), follicle-stimulating hormone (FSH), and oxidative stress index in diabetic male mice.

## 2. Materials and Methods

### Drugs

All chemicals were of analytical grade. MGO, Gal, and met were obtained from Sigma Company (St. Louis, MO), and the Ketamine, and xylazine from Alfasan (the Netherlands).

### Animals and experimental details

This experimental study was performed at the Physiology Research Center, Medical Basic Sciences Research Institute, Ahvaz Jundishapur University of Medical Sciences, Ahvaz, Iran between January and July 2020. Fifty normal adult male NMRI mice, weighing 25-30 gr, were chosen and housed in group cages under standard laboratory conditions (24 ± 2°C, 12-hr light/dark cycle, and humidity 35 ± 5%). The animals used the standard pellet diet and had free access to water. The experiments were performed in accordance with the National Institutes of Health Guide for the Care and Use of Laboratory Animals. Acclimatization took one week. Then animals were then allocated randomly into one of the following five groups (n = 10/each). These groups include:

i. Control (received normal saline)

ii. Diabetic group (received MGO 600 mg/kg/d for 28 days) (10)

iii. Normal group (received GA 50 mg/kg/d for 21 days) (11)

iv. Diabetic treated group (received MGO 600 for 28 days + Gal 50 mg/kg/d for 21 days)

v. Diabetic treated group (received MGO 600 for 28 days + met 200 mg/kg/d for 21 days) (12).

All treatments were administered by gavage (orally). After receiving a daily MGO 600 mg/kg dose, animals with Fasting blood glucose (FBG) ≥ 126 mg/dl were considered diabetic. Gal or met was administered orally for 21 consecutive days after the induction of diabetes.

### Analysis of tissue 

Following an overnight fasting, the animals were anesthetized with ketamine (60 mg/kg) and xylazine (10 mg/kg), blood was taken from the heart, the testis region was cut, and the epididymis was removed for sperm count. The blood samples were centrifuged in 3500 RPM for 10 min and plasma was taken for the next analysis. One of the testes was removed and homogenized with Phosphate buffered saline and supernatant and stored at -70°C for measurement of oxidative stress indices. The other testicles were fixed in 10% formalin and after processing were used for hematoxylin/eosin staining. Then the seminiferous tubule diameters and the height of the epithelia cells were measured with Motic image plus2 software analysis.

### Hormonal assessment and antioxidant enzyme activities

The testosterone, LH, FSH, malondialdehyde (MDA), superoxide dismutase (SOD), and insulin levels were assayed using an enzyme-linked immunosorbent assay (ELISA) kit (Zell Bio Company, Germany). The glucose level was measured with a glucose oxidase commercial kit (Pars Azmoon, Iran). The sperm count was calculated in accordance with the previous report (13).

### Testicular morphology assessment 

The right testicle of the animal was removed for morphological evaluation. Testicular weight, width and length were measured. Testicular volume (TV) was calculated using the following formula: TV = (D${}^{2}$/4×π) × L × K (L = length; D = width, K = 0.9; π = 3.14) (3).

### Sperm assessment

The cauda region of the epididymis of all animals was dissected and transferred into 3-ml of normal saline and cut into small slices. Then, spermatozoa were dispersed into the normal saline and counted with a Neubauer hemocytometer. The sperm count in the white blood cell chambers was counted with a light microscope (Olympus Light Microscope; Olympus Corp., Tokyo, Japan). Data were shown as the number of sperm per milliliter (3).

### Histology assessment

The left testis of each animal was removed and kept in a 10% formalin solution. Six microscopic slides per animal were evaluated for histological features such as vacuolization (empty spaces within the germinal epithelium) and sloughing the germinal epithelium. The average percentage of normal and damaged tubules was also estimated (3). We also considered 150 tubules for each mouse to determine the seminiferous tubules diameters, and the lumen diameter using a Mutic Images software (Plus 3.0).

### Ethical considerations

All experimental procedures were approved by the local ethics committee of Ahvaz Jundishapur University of Medical Sciences IR.AJUMS.ABHC.REC.1398.49.

### Statistical analysis

Data are expressed as mean ± SE. Statistical analysis was completed using SPSS software version 17 (Statistical Package for the Social Sciences, Inc., Chicago, II, USA). Kruskal-Wallis test was used to determine the normality of the variables. Also, the variance was tested for homogeneity. A comparison of the data between the groups was achieved using a one-way analysis of variance (ANOVA) followed by post hoc least significant difference tests. P < 0.05 were considered statistically significant.

## 3. Results

### Fasting blood glucose, insulin levels, and oxidative stress indices

FBG and insulin level in the MGO group were significantly higher than in the control group (p < 0.001), which is the strongest index for diabetes induction; Gal and met administration were associated with significantly lower FBG and insulin level (p < 0.001). Also, the MDA level in the MGO group was significantly higher than the control group (p < 0.001), and treatment with Gal and met was associated with considerably lower values for these parameters (p < 0.001). SOD activity in the MGO group was significantly lower than in the control group (p = 0.02), and treatment with GA (p = 0.002) and met (p = 0.001) was associated with considerably higher in SOD activity (Table I).

### Effect of MGO, Gal, and met on the testicular morphology 

Although the testis width (p = 0.007) and volume (p = 0.01) in the MGO group were significantly smaller than inthe control group, no significant differences were noted in the weight and width of testes between the groups (p > 0.05). Administration of Gal and MGO + Gal was associated with a large width (p = 0.03) and volume of the testis (p = 0.04) compared to the MGO group, while a smaller relationship was not found for the met administration (Table II).

### Sperm parameters analysis and testicular histopathology

**Figure 1:**
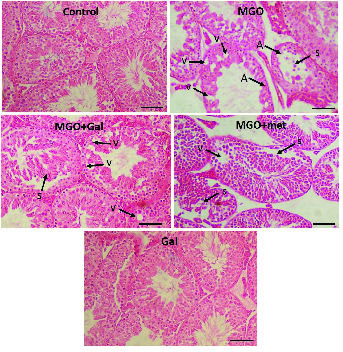
The effect of methylglyoxal (MGO), gallic acid (Gal), and metformin (met) on histological changes in the seminiferous tissue (H&E, Magnification: ×250, n = 10). S: Sloughing of seminiferous epithelium, V: Vacuole, A: Atrophy. Scale bar: 100 µm.

The sperm concentration in the MGO group was significantly lower than in the control group (p < 0.01). The results suggested that treatment with Gal (p < 0.001), MGO + Gal (p < 0.001), and MGO + met (p = 0.03) improved sperm count in comparison to the MGO group. The histological assessment indicated that seminiferous tubule diameters and epithelial height were significantly smaller in the diabetic group in comparison with the control group (p < 0.001). The results suggested that administration of Gal (p < 0.001), MGO + Gal (p < 0.001), and MGO + met (p = 0.008) improved these parameters compared to the MGO group (Table III). Also, many vacuoles were observed in the seminiferous tubule epithelia of the MGO group. The results suggested that treatment with Gal and met can ameliorate MGO-induced histological changes such as in the percentage of normal seminiferous tubules and in their sloughing, vascularization and atrophy (Table IV; Figure 1).

### Effects of Gal on testosterone, LH, and FSH levels 

**Figure 2:**
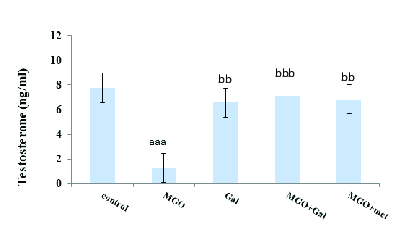
The effect of methylglyoxal (MGO), gallic acid (Gal), and metformin (met) on testosterone. Results are presented as Mean ± SE, n = 10. aaa: P < 0.001 compared with the control group, bb: P = 0.002 and bbb: P < 0.001 compared with the MGO group.

**Figure 3:**
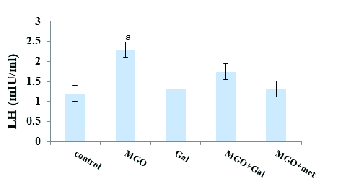
The effect of methylglyoxal (MGO), gallic acid (Gal), and metformin (met) on LH concentration in all groups. Results are presented as Mean ± SE; n = 10. a: P = 0.04 compared with the control group.

**Figure 4:**
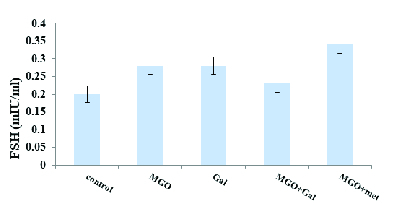
The effect of methylglyoxal (MGO), gallic acid (Gal), and metformin (met) on FSH concentration in all groups. Results are presented as Mean ± SE, n = 10.

Diabetes induction with MGO was associated with significantly lower in testosterone (p < 0.001) (Figure 2) and significantly higher LH (p = 0.04) (Figure 3) but not FSH (Figure 4) in the diabetic group compared to the control group. The results suggested that administration of Gal (p < 0.01), MGO + Gal (p < 0.001), and MGO + met (p = 0.002) in the diabetic group improved testosterone and LH levels but had no beneficial effect on the FSH level.

**Table 1:** Effect of methylglyoxal, gallic acid, and metformin on fasting blood glucose, insulin, and oxidative stress indices


**Groups**	**FBG (mg/dl)**	**Insulin (µIU/ml)**	**MDA (µM)**	**SOD activity (U/ml)**
**Control**	97.17 ± 17.63	4.12 ± 0.839	26.14 ± 5.45	88.03 ± 12.84
**MGO**	194.83 ± 19.32${}^{***}$	8.23 ± 0.993${}^{***}$	71.14 ± 6.23${}^{***}$	77.48 ± 10.49${}^{*}$
**Gal**	98.83 ± 3.061${}^{\#\#\#}$	4.34 ± 0.232${}^{\#\#\#}$	32.63 ± 8.59${}^{\#\#\#}$	89.86 ± 4.24${}^{\#}$
**MGO+ Gal**	98 ± 1.86${}^{\#\#\#}$	6.43 ± 0.369${}^{***\#\#\#!!!}$	34.26 ± 11.04${}^{\#\#\#}$	93.60 ± 3.51${}^{\#\#}$
**MGO + met**	127.50 ± 2.66${}^{***\#\#\#\$\$\$}$	4.27 ± 0.398${}^{\#\#\#}$	28.55 ± 9.40${}^{\#\#\#}$	95.28 ± 1.50${}^{\#\#\#}$
${}^{*}$P = 0.02 and ${}^{***}$P < 0.001 vs Control, ${}^{\#}$P = 0.02, ${}^{\#\#}$P = 0.002, and ${}^{\#\#\#}$P < 0.001 vs MGO, ${}^{\$\$\$}$P < 0.001 MGO + met vs MGO + Gal and Gal, ${}^{!!!}$P < 0.001 MGO + Gal vs MGO + met and Gal. Mean ± SD, One-way ANOVA, and post-hoc LSD test. MGO: Methylglyoxal, Gal: Gallic acid, Met: Metformin, FBG: Fasting blood glucose, MDA: Malondialdehyde, SOD: Superoxide dismutase				

**Table 2:** The effect of methylglyoxal, gallic acid, and metformin on the testicular morphology of male reproductive organ (n = 10)


**Groups**	**Testis weight (mg)**	**Testis length (mm)**	**Testis width (mm)**	**Testis volume (mm${}^{3}$)**
**Control**	96.72 ± 12.71	6.88 ± 0.44	4.66 ± 0.816	108.78 ± 41.900
**MGO**	95.23 ± 20.49	6.85 ± 0.57	3.66 ± 0.516${}^{**}$	66.46 ± 19.65${}^{**}$
**Gal**	84 ± 16.43	7.03 ± 0.68	4.42 ± 0.252${}^{\#}$	97.73 ± 10.25${}^{\#}$
**MGO + Gal**	93.83 ± 9.82	6.95 ± 0.188	4.45 ± 0.400${}^{\#}$	98.68 ± 17.65${}^{\#}$
**MGO + met**	91.83 ± 17.55	7.16 ± 0.40	4.16 ± 0.752	90.07 ± 30.22
Mean ± SD, One-way ANOVA, and post-hoc LSD test. **P < 0.01 vs Control, ${}^{\#}$P < 0.05 vs MGO. MGO: Methylglyoxal, Gal: Gallic acid, Met: Metformin				

**Table 3:** The effect of methylglyoxal, gallic acid, and metformin on seminiferous diameter, epithelium height, and sperm count (×10${}^{6}$)


**Groups**	**Seminiferous diameter (µm)**	**Seminiferous** **height (µm)**	**Sperm count (×10${}^{6}$)**
**Control**	218.2 ± 21.5	72.7 ± 8.6	2.45 ± 0.22
**MGO**	149.7 ± 17.2${}^{***}$	37.5 ± 3.8${}^{***}$	1.28 ± 0.03${}^{***}$
**Gal**	221.3 ± 28.1${}^{\#\#\#}$	73.3 ± 7.9${}^{\#\#\#}$	2.74 ± 0.39${}^{\#\#\#}$
**MGO + Gal**	214.5 ± 19.5${}^{\#\#\#}$	61.3 ± 5.2${}^{\#\#}$	2.16 ± 0.14${}^{\#\#}$
**MGO + met**	203.1 ± 22.4${}^{\#\#}$	58.2 ± 4.9${}^{\#\#}$	1.73 ± 0.08${}^{*\$\$}$
${}^{*}$P = 0.03 and ${}^{***}$P < 0.001 vs control, ${}^{\#\#}$P = 0.008 and ${}^{\#\#\#}$P < 0.001 vs MGO group, ${}^{\$\$}$P = 0.003 gallic acid vs MGO + metformin. Mean ± SD, One-way ANOVA, and post-hoc LSD test. MGO: Methylglyoxal, Gal: Gallic acid, Met: Metformin			

**Table 4:** The effect of methylglyoxal, gallic acid, and metformin on histological changes such as in the percentage of normal, seminiferous tubules, and in their sloughing, vascularization, and atrophy


**Groups**	**Normal (%)**	**Sloughing (%)**	**Vacuolization (%)**	**Atrophy (%)**
**Control**	97.3 ± 0.21	0.6 ± 0.12	2.1 ± 0.17	0.00
**MGO**	32.4 ± 4.2${}^{**}$	11.2 ± 1.9${}^{***}$	48.3 ± 5.2${}^{***}$	8.1 ± 2.1${}^{***}$
**Gal**	97.8 ± 0.14${}^{\#\#}$	0.4 ± 0.07${}^{\#\#\#}$	1.8 ± 0.14${}^{\#\#\#}$	0.00${}^{\#\#\#}$
**MGO + Gal**	89.2 ± 4.8${}^{\#}$	1.5 ± 0.26${}^{*\#\#\#}$	8.9 ± 1.9${}^{\#\#}$	0.4 ± 0.05${}^{**\#\#\#}$
**MGO + met**	84.0 ± 5.3${}^{\#}$	3.4 ± 0.33${}^{**\#\#}$	11.5 ± 2.2${}^{**\#\#}$	1.1 ± 0.22${}^{***\#\#}$
${}^{*}$P = 0.03, ${}^{**}$P < 0.01, and ${}^{***}$P < 0.001 vs. Control, ${}^{\#}$P = 0.02, ${}^{\#\#}$P < 0.01, and ${}^{\#\#\#}$P < 0.001 vs MGO. Mean ± SD, One-way ANOVA, and post-hoc LSD test. MGO: Methylglyoxal, Gal: Gallic acid, Met: Metformin				

## 4. Discussion

The results showed that diabetes is well induced by MGO, as the measured blood glucose and plasma insulin levels clearly indicate this, in agreement with the study of Lee and colleagues (10). According to the present findings, Gal and met administration can cause an improvement in diabetes outcomes. Our findings demonstrated the antidiabetic effect of Gal.

In this study, MGO administration was associated with smaller seminiferous tubule diameters, epithelial height, sperm count, level of testosterone, SOD activity, and testis volume, while the level of LH and malondialdehyde were higher and there was no significant differences in the levels of FSH. Studies on diabetic rats and diabetic men have also confirmed a decline in male reproductive ability (4-6). These studies also showed that male diabetic mice can have diminished testis volume and a lower epididymis sperm count (1, 10). In a study of the oxidative balance of the male reproductive system, it was shown that hyperglycemia can cause oxidative stress and apoptosis (14). Diabetic patients may experience sexual dysfunction (15). Plasma MGO levels are 1.3 times higher than in healthy people (4). Glucose metabolism in testicular cells has unique properties that cause these cells to be damaged in diabetic patients (1, 16). Also, reactive oxygen species produced through hyperglycemia are an important mechanism by which DM affects male reproductive health (1). It was showed that intravenous injection of MGO, inducing type-2 diabetes, can lead to dysfunction of beta cells (5). It was also reported that the incidence of diabetes, hyperglycemia, and pancreatic damage due to MGO in mice (17).

In the present study, Gal administration was associated with larger sperm count, seminiferous tubule diameters, epithelial height, testosterone, and LH level. These findings indicated positive effects of Gal on the reproductive system in diabetic male mice. In regard to the antioxidant effect of Gal, it was concluded that this effect may be related to the antioxidant properties of Gal. In this study, Gal and met seemed to ameliorate the MGO effects. These results are consistent with at study of Gal administration in diabetic rat models that reported an increase in the ability of the male reproductive system (7, 8). In our study, the effect of Gal was equivalent and sometimes better than met; for example GA was associated with a higher testis volume and improved sperm count compared to the MGO group, but met did not demonstrate similar effects. It was reported an effect of met on MGO in patients with type-2 diabetes (18). It was also reported that metformin was effective in reducing glycation (19). Studies have shown that Gal can improve beta cells, and can also be used as a drug to increase insulin secretion (20, 21). In agreement with our study, it was reported that Gal can have antidiabetic potential in *Emblica officinalis* and can be effective in regulating pAkt, PPAR-γ, and Glut4 through its antidiabetic activity. Therefore, they recommended *E. officinalis* as a dietary supplement to protect against diabetes (22). As a result, Gal could be a candidate for diabetes-related changes in the male reproductive system. Yousuf and colleagues reported that advanced glycation end products/receptors for AGEs downstream signaling stimulates reactive oxygen species and decrease the antioxidant status that collectively synthesizes collagen and fibrosis. They showed that concomitant treatment of Gal with glyoxal inhibited these changes and improved the fibrotic effects of glyoxal in rats (23). It was showed that Gal can improve insulin resistance in type-2 diabetic rats. Gal also increased the glucose uptake and translocation of insulin-stimulated glucose transporters, decreases FBG, and improved the glucose tolerance test (24). Gal may be of benefit in reproductive health protection by decreasing diabetic-based testicular damage (25).

Surprisingly, the lower testis weight in the MGO group compared to the control group was not statistically significant, but the testis volume in the MGO group was much lower than in the control group. Decreased TV is associated with lower testicular function (< 12 cm${}^{3}$). It was reported that the conventional sperm parameters (concentration, motility, and morphology) and endocrine function (gonadotropins and testosterone serum concentrations) were altered with reduced TV (26). Daily sperm production and TV are significantly reduced in older men (27). Therefore, TV represents an androgen index that is even better than a single measurement of testosterone (28).

This study had some limitations: first, we did not have enough budget to investigate cell signaling pathways, and second, we did not conduct any genetics experiments. Further research is needed on genetic and cellular evaluations.

## 5. Conclusion

The study found that MGO induced DM by promoting oxidative stress, reducing the abilities of the male reproductive system and fertility. Gal and met were associated with higher SOD activity and testosterone levels and lower LH and MDA levels, which improve the male reproductive system. Gal and met also appeared to have a positive effect on the tissue of the testis. Thus, it seems from the results that Gal and met improved the diabetes-related changes in the reproductive system by inhibiting oxidative stress in the MGO-induced diabetic model, and stopping the production of free radicals. The study also found that the GA effect was equal to and even better than met; for example, Gal was associated with larger TV and sperm count compared to the MGO group, but met was not. The study's findings suggest that Gal could be a candidate for reducing diabetes-related changes in the male reproductive system.

##  Conflict of Interest

The authors declare no conflict of interest.
